# A new subterranean species of *Pseudocrangonyx* from China with an identification key to all species of the genus (Crustacea, Amphipoda, Pseudocrangonyctidae)

**DOI:** 10.3897/zookeys.647.11192

**Published:** 2017-01-23

**Authors:** Shuangyan Zhao, Zhonge Hou

**Affiliations:** 1 Institute of Zoology, Chinese Academy of Sciences, Beijing 100101, China; 2 College of Life Sciences, Hebei University, Baoding, Hebei 071002, China

**Keywords:** cave, COI distance, molecular phylogeny, Pseudocrangonyx, taxonomy

## Abstract

A subterranean species of *Pseudocrangonyx elegantulus* Hou, **sp. n.** is described from caves of Wulongdong National Forest Park in Henan Province, China. *Pseudocrangonyx elegantulus* is characterized by both male and female with calceoli on antenna II; urosomite III dorsal margin without armature; uropod III with peduncle 0.30 times as long as outer ramus and terminal article of the outer ramus a little shorter than adjacent spines; telson cleft 0.27 of its length. Phylogenetic analysis based on 28S and COI sequences supported the species distinctness. A key to the genus *Pseudocrangonyx* with 22 species and a map of their distributions are provided.

## Introduction

The genus *Pseudocrangonyx* was established by Akatsuka and Komai in 1922, including 21 described species that are widely distributed in subterranean freshwaters or springs of Japan, the Korean peninsula, eastern China, and the Far East of Russia (Labay 2001, Sidorov and Gontcharov 2013, Tomikawa et al. 2016). The genus exhibits typical subterranean adaptive morphology in the loss of eyes and pigmentation, elongated appendages, and vestigialization of dorsal armature on urosomites (Sidorov and Gontcharov 2013).

To date, 13 species are known from the Far East of Russia, including *Pseudocrangonyx bohaensis* (Derzhavin, 1927), *Pseudocrangonyx levanidovi* Birstein, 1955, *Pseudocrangonyx camtschaticus* Birstein, 1955, *Pseudocrangonyx birsteini* Labay, 1999, *Pseudocrangonyx relicta* Labay, 1999, *Pseudocrangonyx susanaensis* Labay, 1999, *Pseudocrangonyx korkishkoorum* Sidorov, 2006, *Pseudocrangonyx febras* Sidorov, 2009, *Pseudocrangonyx elenae* Sidorov, 2011, *Pseudocrangonyx kseniae* Sidorov, 2012, *Pseudocrangonyx holsingeri* Sidorov & Gontcharov, 2013, *Pseudocrangonyx sympatricus* Sidorov & Gontcharov, 2013, and *Pseudocrangonyx tiunovi* Sidorov & Gontcharov, 2013. Four species have been described from Japan, *Pseudocrangonyx kyotonis* Akatsuka & Komai, 1922, *Pseudocrangonyx shikokunis* Akatsuka & Komai, 1922, *Pseudocrangonyx yezonis* Akatsuka & Komai, 1922, and *Pseudocrangonyx gudariensis* Tomikawa & Sato, 2016. One species was recorded in South Korea, *Pseudocrangonyx coreanus* Uéno, 1966. Three species are known from China, *Pseudocrangonyx manchuricus* Oguro, 1938, *Pseudocrangonyx asiaticus* Uéno, 1934, and *Pseudocrangonyx cavernarius* Hou & Li, 2003. The genus shows a broad distribution along the northern Asia-Pacific margins. This is expected to be related to the land-bridges formed with the fluctuations of sea level. However, the evolutionary history of the genus *Pseudocrangonyx* was poorly discussed, and most studies focused on species revision and discovery.

**Figure 1.**
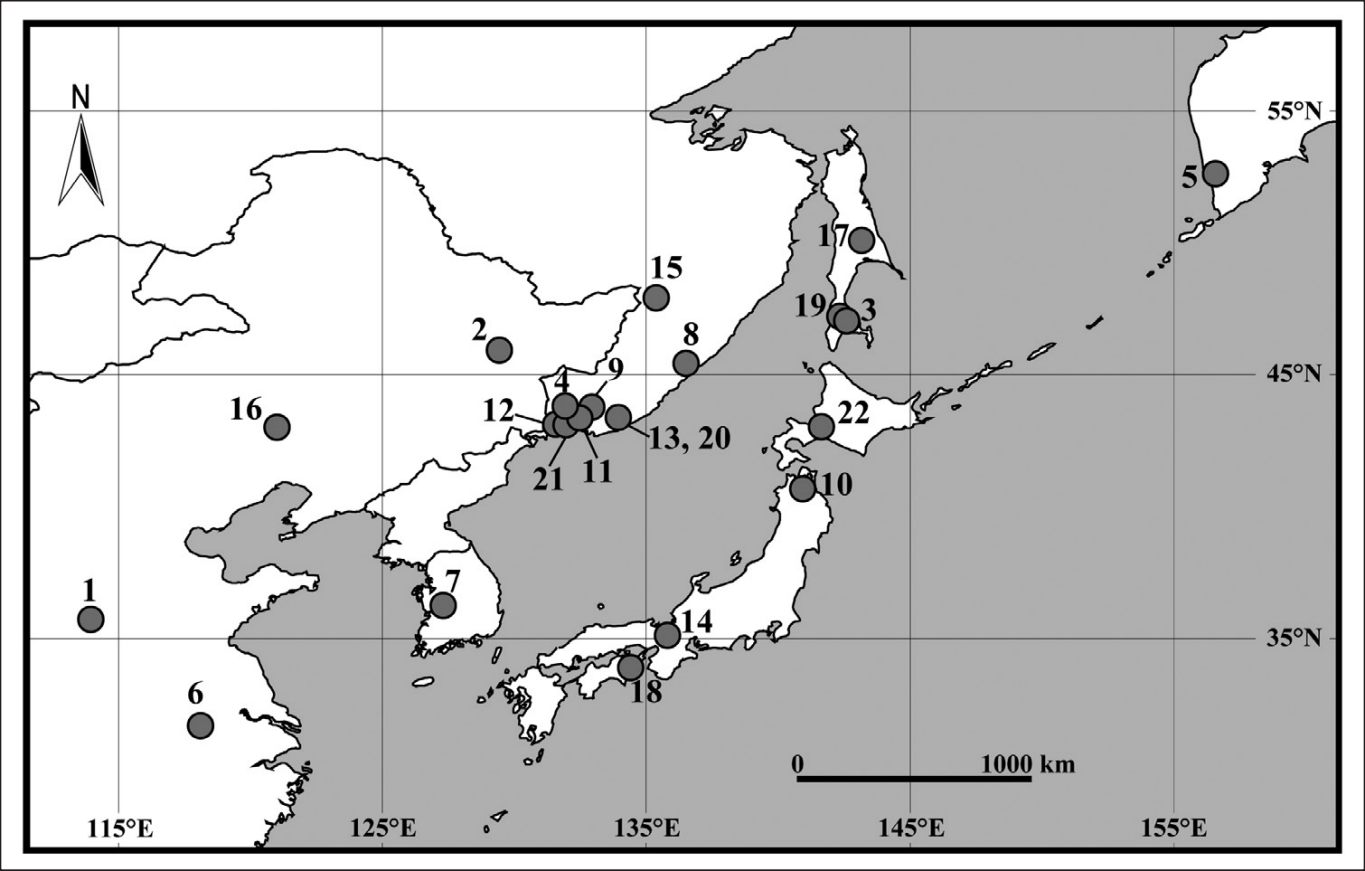
Distribution map of *Pseudocrangonyx* species. **1** *Pseudocrangonyx elegantulus* sp. n. **2** *Pseudocrangonyx asiaticus* Uéno, 1934 **3** *Pseudocrangonyx birsteini* Labay, 1999 **4** *Pseudocrangonyx bohaensis* (Derzhavin, 1927) **5** *Pseudocrangonyx camtschaticus* Birstein, 1955 **6** *Pseudocrangonyx cavernarius* Hou & Li, 2003 **7** *Pseudocrangonyx coreanus* Uéno, 1966 **8** *Pseudocrangonyx elenae* Sidorov, 2011 **9** *Pseudocrangonyx febras* Sidorov, 2009 **10** *Pseudocrangonyx gudariensis* Tomikawa & Sato, 2016 **11** *Pseudocrangonyx holsingeri* Sidorov & Gontcharov, 2013 **12** *Pseudocrangonyx korkishkoorum* Sidorov, 2006 **13** *Pseudocrangonyx kseniae* Sidorov, 2012 **14** *Pseudocrangonyx kyotonis* Akatsuka & Komai, 1922 **15** *Pseudocrangony levanidovi* Birstein, 1955 **16** *Pseudocrangonyx manchuricus* Oguro, 1938 **17** *Pseudocrangonyx relicta* Labay, 1999 **18** *Pseudocrangonyx shikokunis* Akatsuka & Komai, 1922 **19** *Pseudocrangonyx susanaensis* Labay, 1999 **20** *Pseudocrangonyx sympatricus* Sidorov & Gontcharov, 2013 **21** *Pseudocrangonyx tiunovi* Sidorov & Gontcharov, 2013 **22** *Pseudocrangonyx yezonis* Akatsuka & Komai, 1922.

During a field survey of freshwater amphipods in Henan Province, China, three species were found, including two epigean freshwater gammarids, *Gammarus preciosus* Wang et al., 2009 and *Gammarus monticellus* Hou et al., 2014, and one cave *Pseudocrangonyx* species new to science. In this paper, the fourth species, *Pseudocrangonyx elegantulus* sp. n., is described and illustrated. In addition, the phylogenetic position of the new species within *Pseudocrangonyx* was estimated using nuclear 28S rRNA and mitochondrial cytochrome *c* oxidase subunit I (COI) sequence data. The distributions of all 22 species of the genus *Pseudocrangonyx* are presented in Figure 1, where only type localities are used for *Pseudocrangonyx elegantulus*, *Pseudocrangonyx korkishkoorum*, *Pseudocrangonyx febras*, *Pseudocrangonyx cavernarius*, *Pseudocrangonyx tiunovi*, *Pseudocrangonyx holsingeri*, *Pseudocrangonyx sympatricus*, *Pseudocrangonyx gudariensis*, *Pseudocrangonyx elenae*, *Pseudocrangonyx kseniae*, *Pseudocrangonyx manchuricus*, and *Pseudocrangonyx asiaticus*, and others are based on the published paper (Sidorov 2006). A key to world species of the genus *Pseudocrangonyx* is provided.

## Materials and methods

### Morphological observations

The specimens were collected by sweeping various groundwater environments with a fine-meshed hand net. Samples preserved in 95% ethanol in the field, then deposited in a -20°C refrigerator for long-term preservation. The body length was recorded by holding the specimen straight and measuring the distance along the dorsal side of the body from the base of the first antenna to the base of the telson. All dissected appendages were mounted on slides according to the methods described by Holsinger (1967). Appendages were drawn using a Leica DM2500 compound microscope equipped with a drawing tube. All types and other material are lodged in the Institute of Zoology, Chinese Academy of Sciences
(IZCAS), Beijing.

### DNA sequencing and phylogenetic analyses

Genomic DNA was extracted from appendages of the *Pseudocrangonyx* specimen using a TIANamp Genomic DNA Kit (TIANGEN). The fragments of 28S and COI were amplified and sequenced following published protocols (Hou et al. 2007). The new sequences and reference sequences downloaded from GenBank were aligned using MAFFT v.7.304 (Katoh and Standley 2016). In total, 29 samples of 14 *Pseudocrangonyx* species were used in molecular phylogenetic analyses (Table 1). There are seven species from Russian Far East including *Pseudocrangonyx febras*, *Pseudocrangonyx holsingeri*, *Pseudocrangonyx korkishkoorum*, *Pseudocrangonyx kseniae*, *Pseudocrangonyx susanaensis*, *Pseudocrangonyx sympatricus*, and *Pseudocrangonyx tiunovi*, six species from Japan including *Pseudocrangonyx yezonis* and *Pseudocrangonyx gudariensis*, and four newly described species (Tomikawa et al. 2016) and *Pseudocrangonyx elegantulus* sp. n. from China. Three crangonyctoid species were selected as outgroup taxa: *Crymostygius thingvallensis* Kristjánsson & Svavarsson, 2004, *Crangonyx floridanus* Bousfield, 1963, and *Crangonyx pseudogracilis* Bousfield, 1958.

**Table 1.** Samples used for the phylogenetic analyses. The locality information is accompanied by sequence accession numbers. Species names marked with an asterisk were obtained from Tomikawa et al. (2016).

Species	Voucher	Locality	28S	COI
*Pseudocrangonyx elegantulus* sp. n.	1602	Wulongdong National Forest Park, Linzhou, Henan, China	KY436646	KY436647
*Pseudocrangonyx* sp6*	G1298	Gujo, Gifu, Japan	LC171545	LC171546
*Pseudocrangonyx* sp6*	G1297	Gujo, Gifu, Japan	LC171541	LC171542
*Pseudocrangonyx* sp5*	G1296	Kami, Kochi, Japan	LC171537	LC171538
*Pseudocrangonyx* sp5*	G1295	Kami, Kochi, Japan	LC171533	LC171534
*Pseudocrangonyx* sp5*	G1294	Seiyo, Ehime, Japan	LC171529	LC171530
*Pseudocrangonyx* sp5*	G1271	Takamatsu, Kagawa, Japan	LC171502	LC171503
*Pseudocrangonyx gudariensis*	NSMT-Cr24605	Aomori, Aomori, Japan	LC171498	LC171499
*Pseudocrangonyx* sp3*	G406	Taga, Shiga, Japan	LC171495	–
*Pseudocrangonyx* sp3*	G405	Taga, Shiga, Japan	LC171491	LC171492
*Pseudocrangonyx* sp3*	G404	Taga, Shiga, Japan	LC171488	–
*Pseudocrangonyx* sp5*	G402	Matsue, Shimane, Japan	LC171485	LC171486
*Pseudocrangonyx* sp5*	G401	Ota, Shimane, Japan	LC171481	LC171482
*Pseudocrangonyx holsingeri*	S49	Steklajnuha, Primory, Russia	KJ871679	KF153111
*Pseudocrangonyx* sp2*	G1283	Niimi, Okayama, Japan	LC171525	LC171526
*Pseudocrangonyx* sp2*	G1278	Mine, Yamaguchi, Japan	LC171510	LC171511
*Pseudocrangonyx* sp2*	G1277	Mine, Yamaguchi, Japan	LC171506	LC171507
*Pseudocrangonyx yezonis*	G1280	Mukawa, Hokkaido, Japan	LC171518	LC171519
*Pseudocrangonyx yezonis*	G1279	Daisen, Akita, Japan	LC171514	LC171515
*Pseudocrangonyx korkishkoorum*	B1	Barabashevka, Primory, Russia	KJ871678	KF153107
*Pseudocrangonyx korkishkoorum*	N2	Narva, Primory, Russia	KJ871677	KF153106
*Pseudocrangonyx korkishkoorum*	N1	Narva, Primory, Russia	KJ871676	KF153105
*Pseudocrangonyx korkishkoorum*	B3	Barabashevka, Primory, Russia	–	KF153109
*Pseudocrangonyx korkishkoorum*	B2	Barabashevka, Primory, Russia	–	KF153108
*Pseudocrangonyx kseniae*	S66	Kievka, Primory, Russia	KJ871675	KF153115
*Pseudocrangonyx tiunovi*	S13	Vladivostok, Primory, Russia	KJ871674	KF153110
*Pseudocrangonyx febras*	S23	Arsenyevka, Primory, Russia	–	KF153114
*Pseudocrangonyx susunaensis*	S32	Yuzhno-Sakhalinsk, Sakhalin, Russia	–	KF153113
*Pseudocrangonyx sympatricus*	S67	Kievka, Primory, Russia	–	KF153112
*Crangonyx floridanus*	G1322	Chiba, Chiba, Japan	LC171549	LC171550
*Crangonyx pseudogracilis*	–	–	EF522940	EF570296
*Crymostygius thingvallensis*	–	–	HQ286019	HQ286032

The best-fit partitioning schemes and nucleotide substitution models were selected using PartitionFinder v.1.1.0 on the Bayesian criterion (Lanfear et al. 2012). The COI data were partitioned into first, second, and third codon positions with TrN+I+G, TrNef+I+G, and TrN+G models, respectively. The best model for 28S was GTR+G. Therefore, a four-partition scheme was used in the following analyses.

Phylogenetic relationships were inferred using maximum parsimony (MP), maximum likelihood (ML) and Bayesian inference (BI) on single gene and concatenated sequences. MP analysis and bootstrap evaluation were performed using PAUP* 4.0b10 (Swofford 2002) with tree bisection reconnection swapping algorithm. ML phylogenies were conducted using RAxML v.8.2.7 (Stamatakis 2014) with 1000 rapid bootstrap replicates followed by a thorough tree search. Bayesian analyses were carried out using MrBayes v.3.2.1 (Ronquist et al. 2012), implementing two independent runs of five million generations. The convergence was checked using Tracer v.1.5 (Rambautand Drummond 2009) and the first 25% trees were discarded as burn-in.

## Taxonomy

### Family Pseudocrangonyctidae Holsinger, 1989

#### Genus Pseudocrangonyx

Taxon classificationAnimaliaAmphipodaPseudocrangonyctidae

Akatsuka & Komai, 1922

##### Type species.

*Pseudocrangonyx shikokunis* Akatsuka & Komai, 1922.

#### Pseudocrangonyx elegantulus

Taxon classificationAnimaliaAmphipodaPseudocrangonyctidae

Hou sp. n.

http://zoobank.org/702B105F-271E-47F0-BCC9-7B12114A6102

**Figure 2.**
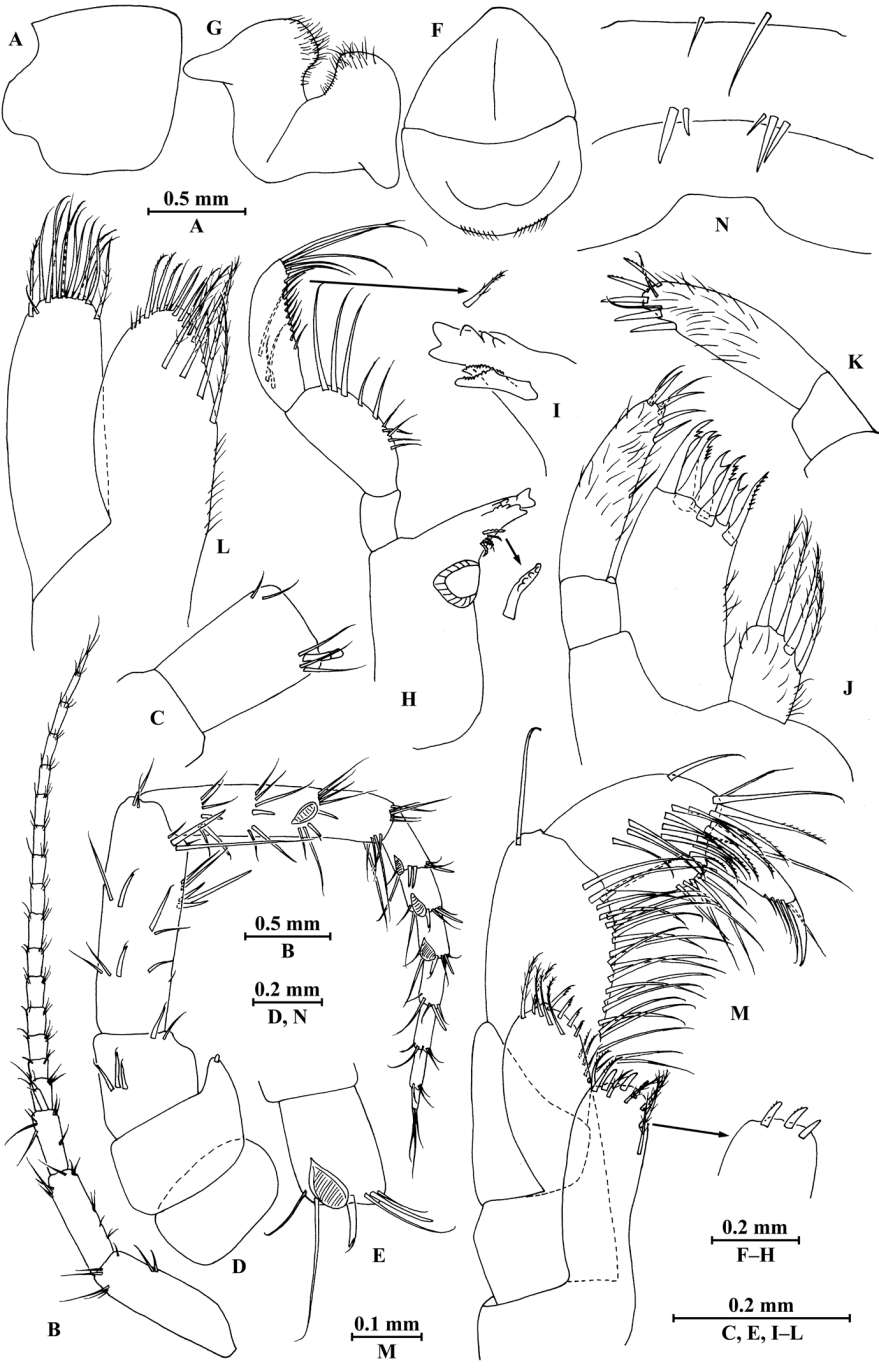
*Pseudocrangonyx elegantulus* sp. n., female holotype, from Henan, China. **A** head **B** antenna I **C** aesthetascs of antenna I **D** antenna II **E** calceoli of antenna II **F** upper lip **G** lower lip **H** left mandible **I** incisor of right mandible **J** left maxilla I **K** palp of right maxilla I **L** maxilla II **M** maxilliped **N** urosomites (dorsal view).

**Figure 3.**
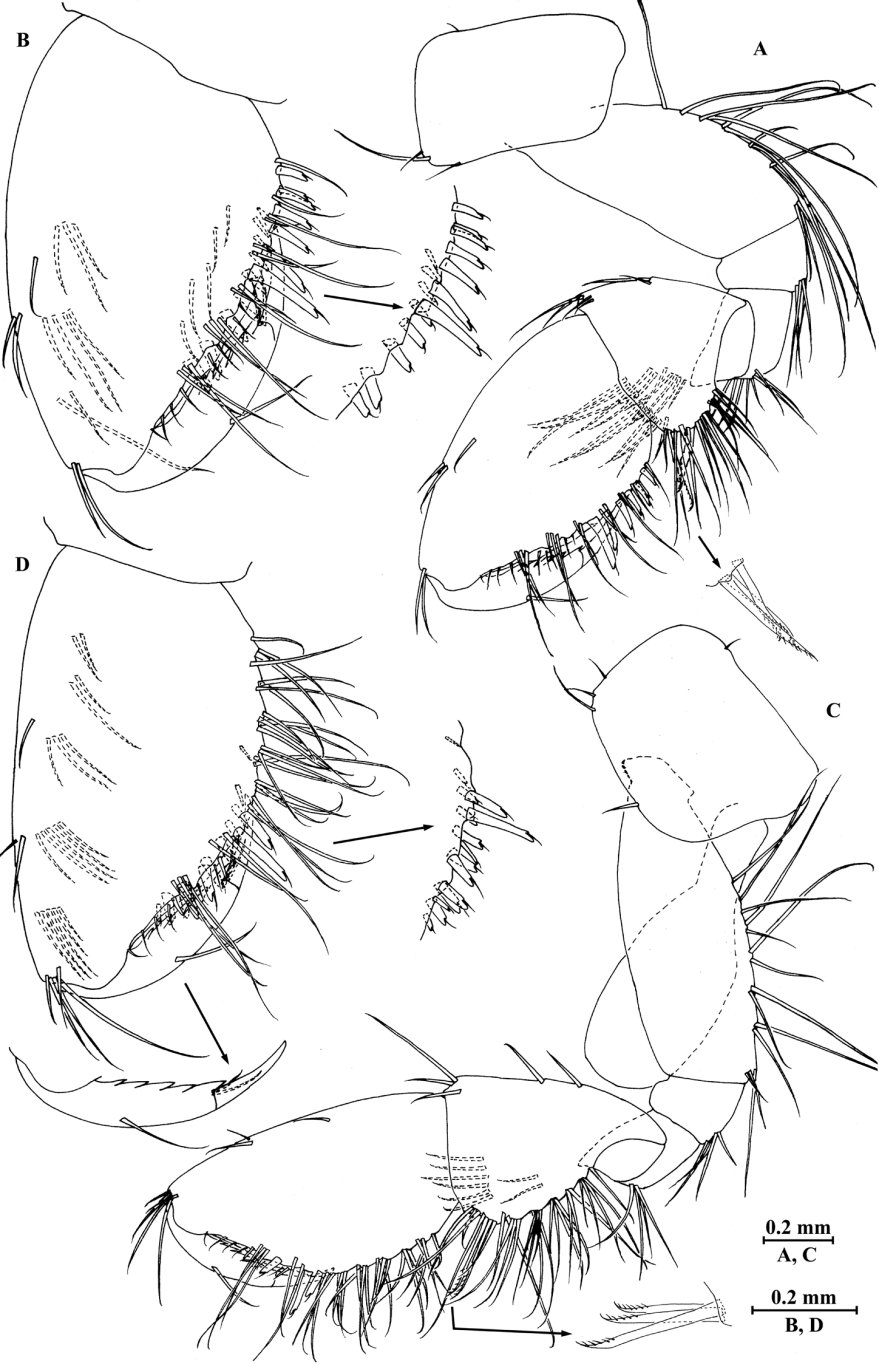
*Pseudocrangonyx elegantulus* sp. n., female holotype. **A** gnathopod I **B** propodus of gnathopod I **C** gnathopod II **D** propodus of gnathopod II.

**Figure 4.**
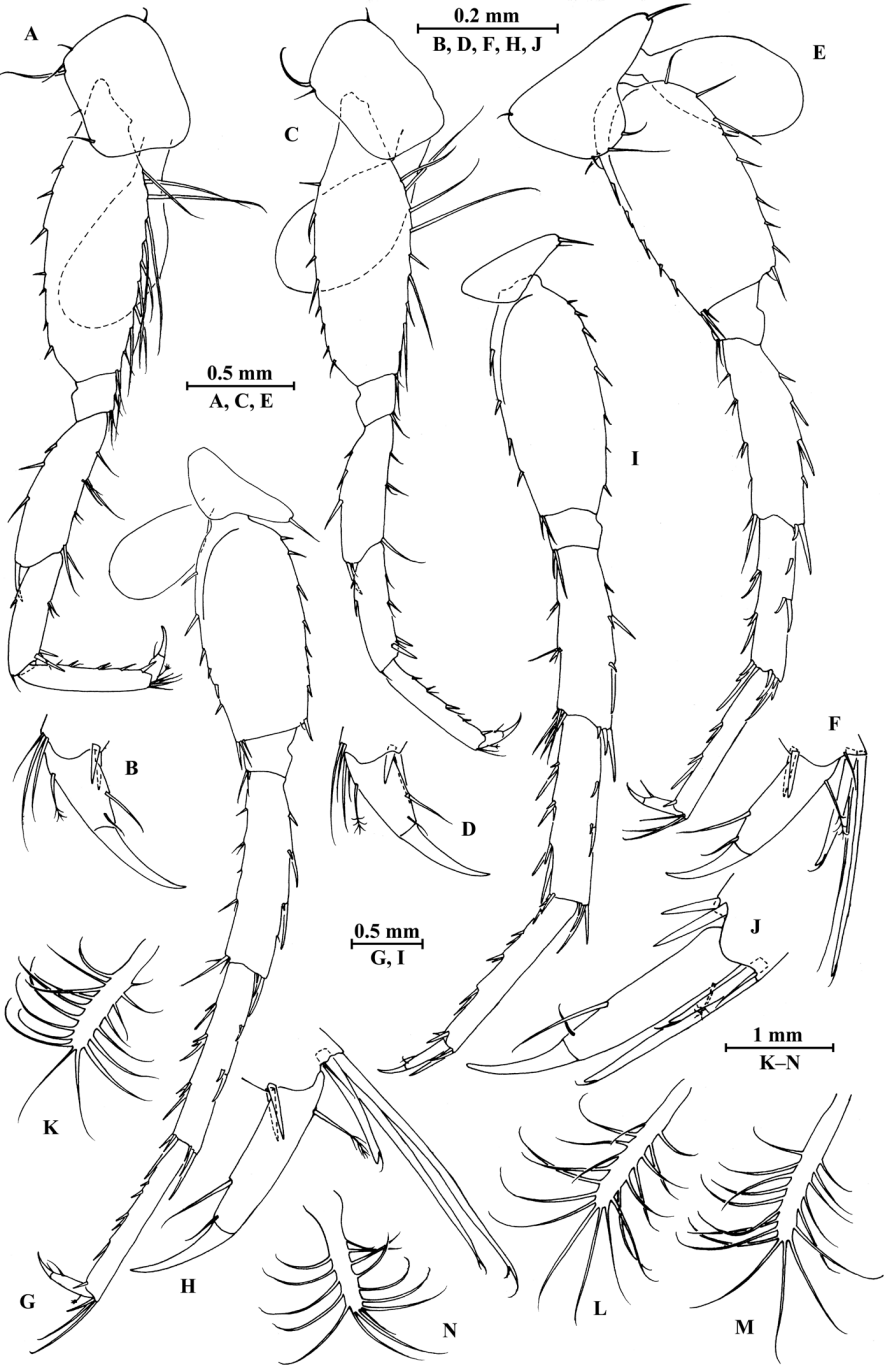
*Pseudocrangonyx elegantulus* sp. n., female holotype. **A** pereopod III **B** dactylus of pereopod III **C** pereopod IV **D** dactylus of pereopod IV **E** pereopod V **F** dactylus of pereopod V **G** pereopod VI **H** dactylus of pereopod VI **I** pereopod VII **J** dactylus of pereopod VII **K** oostegite of gnathopod II **L** oostegite of pereopod III **M** oostegite of pereopod IV **N** oostegite of pereopod V.

**Figure 5.**
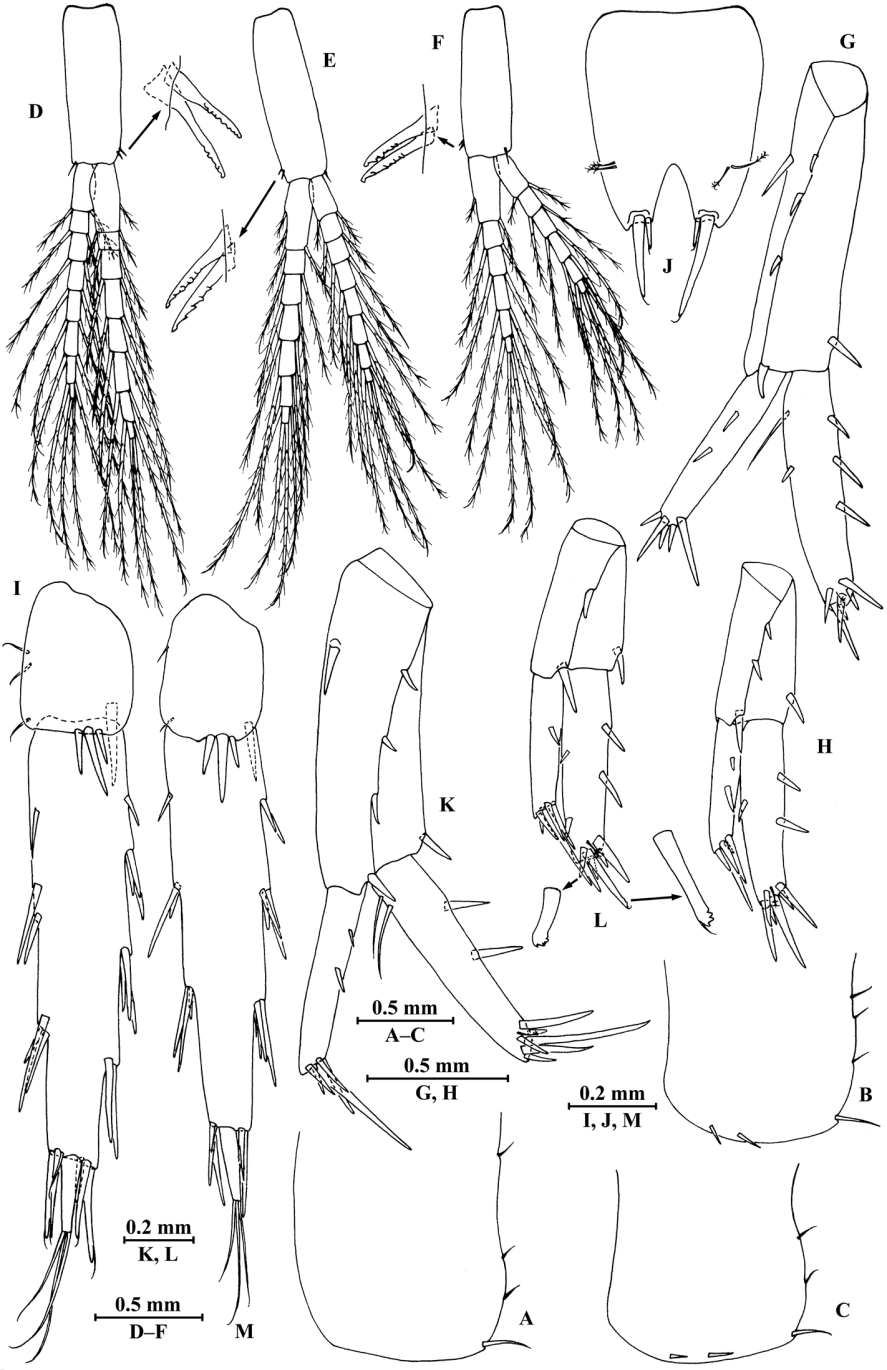
*Pseudocrangonyx elegantulus* sp. n., **A–J** female **K–M** male **A** epimeral plate I **B** epimeral plate II **C** epimeral plate III **D** pleopod I **E** pleopod II **F** pleopod III **G** uropod I **H** uropod II **I** uropod III **J** telson **K** uropod I **L** uropod II **M** uropod III.

**Figure 6.**
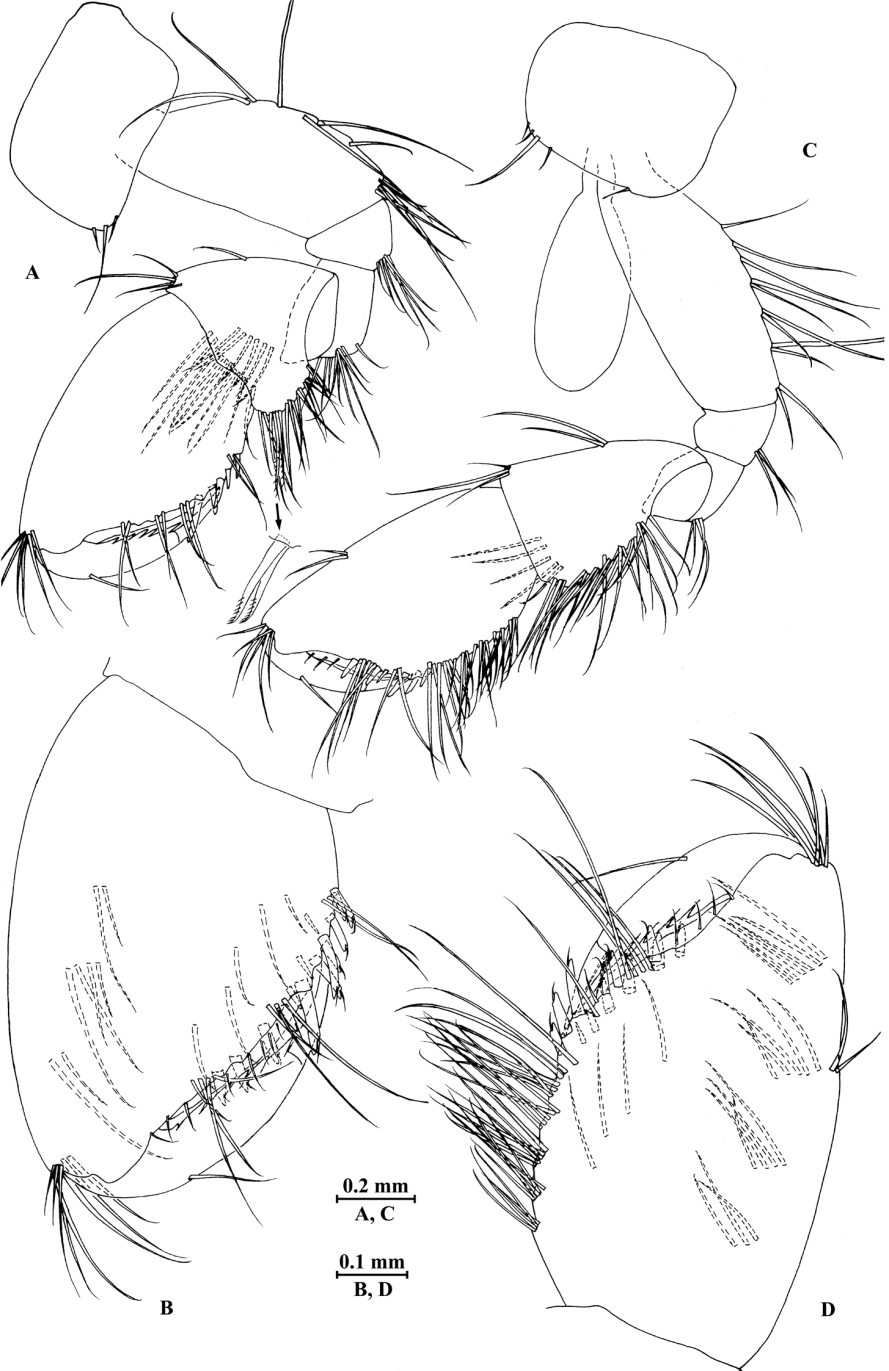
*Pseudocrangonyx elegantulus* sp. n., male paratype. **A** gnathopod I **B** propodus of gnathopod I **C** gnathopod II **D** propodus of gnathopod II.

**Figure 7.**
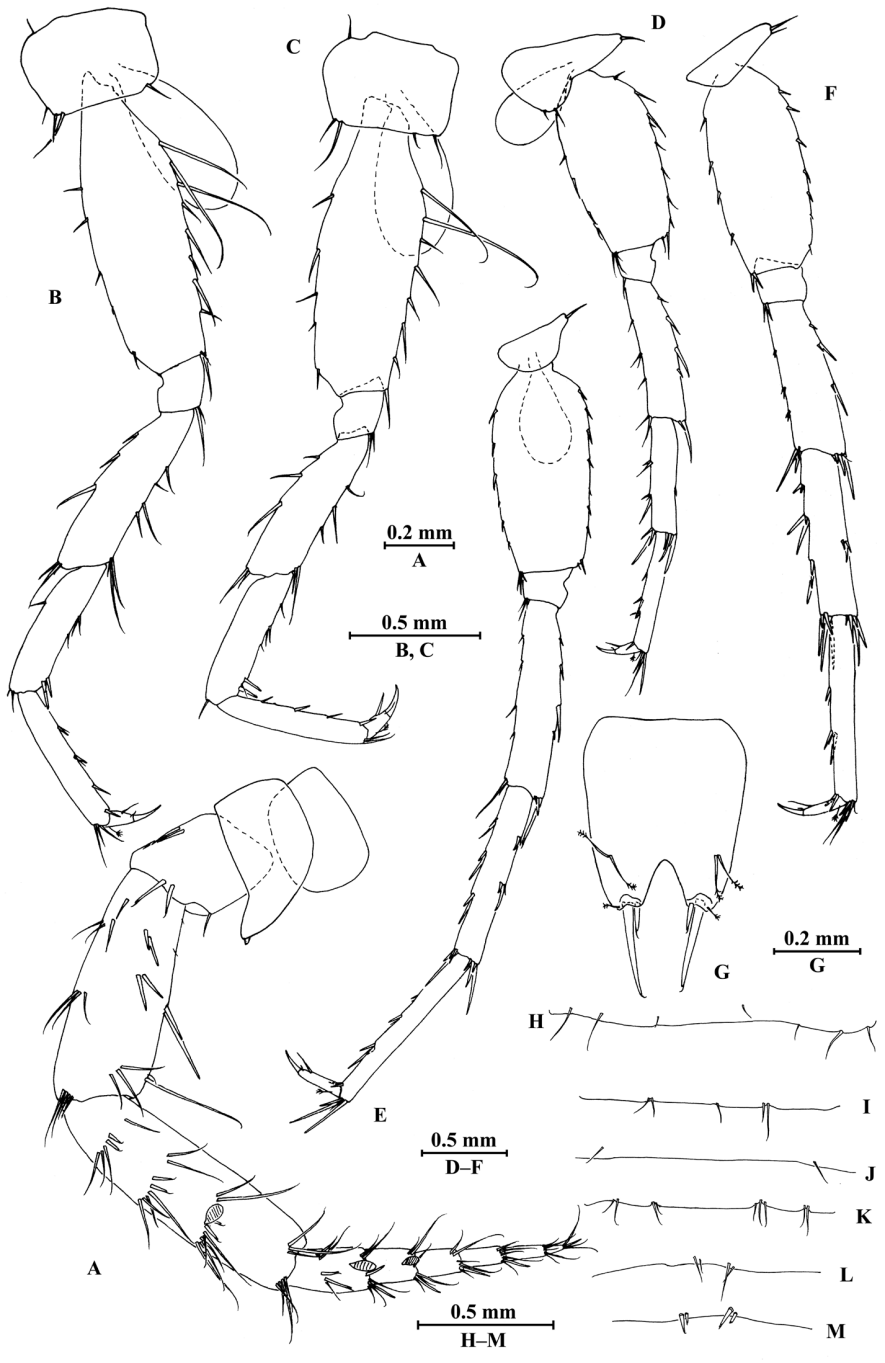
*Pseudocrangonyx elegantulus* sp. n., male paratype. **A** antenna II **B** pereopod III **C** pereopod IV **D** pereopod V **E** pereopod VI **F** pereopod VII **G** telson **H** pereonite VII (dorsal view) **I–K** pleonites (dorsal view) **L, M** urosomites I, II (dorsal view).

Figs 2 , 3 , 4 , 5 , 6 , 7

##### Material examined.

Holotype: female (IZCAS-I-A1602-1), 7.5 mm, Wulongdong National Forest Park (113.943°E, 35.716°N), altitude 770 m, Wulong Town, Linzhou City, Henan Province, China, June 19, 2014, collected by Y. Li and J. Liu. Paratype: male (IZCAS-I-A1602-2), 6.3 mm, same data as holotype.

##### Etymology.

The specific name is from Latin *elegantulus* (elegant), in reference to the peculiar shape; adjectival, masculine.

**Diagnosis.** Female larger than male; eyes absent; lateral cephalic lobe rounded; inferior antennal sinus indistinct; both male and female with calceoli on antenna II; coxal gills present on gnathopod II and pereopods III–VI; sternal gills absent; epimeral plate I without armature on distal margin; urosomite III dorsal margin without armature; uropod I peduncle with one basofacial spine; inner ramus of male uropod II with two serrate and four simple robust terminal spines accompanied by one seta; uropod III peduncle 0.30 times as long as outer ramus and terminal article of the outer ramus a little shorter than adjacent spines.

##### Description of holotype female

(IZCAS-I-A1602-1), 7.5 mm.

**Head.** (Fig. 2A): eyes absent; lateral cephalic lobe rounded; inferior antennal sinus indistinct.

*Antenna I* (Fig. 2B, C): peduncle articles 1–3 in length ratio 1.0 : 0.7 : 0.4, with distal setae; flagellum with 16 articles, articles 3–15 with aesthetascs; accessory ﬂagellum with two articles, subequal to the first article of primary flagellum; both primary and accessory ﬂagella with short distal setae.

*Antenna II* (Fig. 2D, E): peduncle articles 3–5 in length ratio 1.0 : 2.2 : 2.9, with spines accompanied by setae; flagellum with seven articles, with one or two spines and setae on first three articles and with setae on the rest articles; calceoli of crangonyctid type, present on peduncular article 5 and first three flagellum articles; rod-like structures accompanied with setae on first four flagellum articles.

*Upper lip* (Fig. 2F): ventral margin rounded, bearing fine setae.

*Mandible* (Fig. 2H, I): incisor of left mandible with five teeth; lacinia mobilis with five teeth; spine row with five serrated spines; articles 1–3 of palp in length ratio 1.0 : 2.2 : 2.4, second article with ten marginal setae, article 3 with three B-setae, ten D-setae and five E-setae apically; incisor of right mandible with five teeth, lacinia mobilis bifurcate, with small teeth.

*Lower lip* (Fig. 2G): inner lobes absent, outer lobes covered with thin setae.

*Maxilla I* (Fig. 2J, K): asymmetrical, left inner plate with four plumose setae; outer plate with seven serrated apical spines; second article of left palp densely setose, with two simple setae and four slender spines apically; second article of right palp with five spines and two slender setae.

*Maxilla II* (Fig. 2L): inner plate with four plumose facial setae in an oblique row; inner and outer plates with long setae apically.

*Maxilliped* (Fig. 2M): inner plate with three stout apical spines, two serrated setae, and five plumose setae; outer plate bearing four setae, four serrated spines and five plumose setae apically; palp 4-articulate, articles 1–2 in length ratio 0.7 : 1, article 2 with a row of simple setae on interior side and one simple seta on exterior side; article 4 hooked, with five setae at hinge of unguis.

**Pereon.** *Gnathopod I* (Fig. 3A, B): coxal plate bearing one fine seta on proximal margin and three setae on anterodistal corner, 1.7 times as wide as deep; basis with long setae on posterior margin, anterior margin bare; merus bearing setae on posterodistal corner; carpus as long as wide, approximately 0.5 times as long as propodus, bearing three clusters of setae along posterior margin, two clusters of setae on anterior margin, and three pectinate setae on posterodistal corner; propodus pear-shaped, palm margin with 16 robust spines, some distally notched; dactylus with one seta on anterior margin and two setae at hinge of unguis, posterior margin dentate.

*Gnathopod II* (Fig. 3C, D): coxal plate bearing one fine seta on proximal margin, three setae on anterodistal corner and one seta on distal margin; basis with setae on posterior margin, anterior margin bare; merus bearing setae on posterodistal corner; carpus 1.5 times as long as wide, approximately 0.7 times as long as propodus, bearing seven clusters of setae along posterior margin and three pectinate setae on posterodistal corner; propodus stout, palm margin with 14 distally notched spines; dactylus with one seta on anterior margin and two setae at hinge of unguis, posterior margin dentate.

*Pereopod III* (Fig. 4A, B): coxal plate bearing four setae on anterior margin and two setae on distal margin, 1.4 times as wide as deep; basis with seven setae along anterior margin and long setae on posterior margin; merus, carpus, and propodus in length ratio 1.0 : 0.7 : 0.8; merus with three spines on anterior margin and four clusters of setae on posterior margin, anterodistal corner with one spine; carpus with one fine seta on anterior margin and two setae on posterior margin, anterodistal corner with one seta and posterodistal corner with two spines accompanied with one seta; dactylus with one plumose seta on anterior margin, one seta on posterior margin, and one seta at hinge of unguis.

*Pereopod IV* (Fig. 4C, D): similar to pereopod III; coxal plate bearing three setae on anterior margin, 1.6 times as wide as deep; merus, carpus, and propodus in length ratio 1.0 : 0.9 : 1.0.

*Pereopod V* (Fig. 4E, F): coxal plate irregular, anterior lobe larger than posterior lobe, bearing four setae and one seta on anterior and posterior lobes, respectively; basis with setae on anterior and posterior margins, respectively; merus, carpus, and propodus in length ratio 1.0 : 0.9 : 0.9; merus and carpus with spines accompanied by setae on both margins; dactylus with one plumose seta on posterior margin, one seta on anterior margin, and one seta at hinge of unguis.

*Pereopod VI* (Fig. 4G, H): coxal plate similar to that of pereopod V, with smaller anterior lobe, bearing one seta on posterior lobe; basis with setae on anterior and posterior margins; merus, carpus, and propodus in length ratio 1.0 : 1.0 : 0.9; merus and carpus with spines accompanied by setae on both margins; dactylus with one plumose seta on posterior margin, one seta on anterior margin, and one seta at hinge of unguis.

*Pereopod VII* (Fig. 4I, J): coxal plate subtriangular, with two setae on posteroproximal corner; basis with setae on anterior and posterior margins; merus, carpus, and propodus in length ratio 1.0 : 1.1 : 1.1; merus and carpus with spines accompanied by setae on both margins; dactylus with one plumose seta on posterior margin, one seta on anterior margin, and one seta at hinge of unguis.

*Coxal gills*: present on gnathopod II and pereopods III–VI; sternal gills absent.

*Oostegite* (Fig. 4K–N): narrow, present on gnathopod II and pereopods III–V, with marginal setae.

**Pleon.** *Epimeral plates* (Fig. 5A–C): plate I distally rounded, bearing three fine setae on posterior margin and one seta on posterodistal corner, distal margin without armature; plate II with two spines on distal margin and three fine setae on posterior margin, posterodistal corner rounded with one seta; plate III with two spines on distal margin and two fine setae on posterior margin, posterodistal corner rounded with one seta.

*Pleopods I–III* (Fig. 5D–F): similar, peduncle with two retinacula on interior side and one fine seta on exterior distal corner; outer ramus shorter than inner ramus, both inner and outer rami fringed with plumose setae.

**Urosome.** *Urosomites* (Fig. 2N): urosomite I with two setae on dorsal margin; urosomite II with two spines on left side and two spines accompanied by one seta on right side; urosomite III dorsal margin without armature.

*Uropods I–III* (Fig. 5G–I): uropod I peduncle with one basofacial spine, with three spines on exterior side, interior and exterior distal corners with one spine respectively; inner ramus approximately 0.77 times as long as peduncle, with three spines on interior side, one seta and one spine on exterior side, and six terminal spines accompanied by one seta; outer ramus approximately 0.76 times of inner ramus, with two spines on exterior side and five terminal spines. Uropod II half shorter, peduncle bearing two spines on exterior side and one spine on each distal corner; inner ramus with two spines on interior side, one spine on exterior side, and six terminal spines accompanied by one seta; outer ramus approximately 0.71 times of inner ramus, with two spines on exterior side and five terminal spines. Uropod III with peduncle 0.30 times as long as outer ramus, with one dorsal and three ventral robust spines; inner ramus absent; outer ramus 2-articulate, first article of outer ramus with stiff spines on interior and exterior sides, terminal article 0.19 times of the first article, with three distal setae, a little shorter than adjacent spines.

*Telson* (Fig. 5J): 1.2 times as long as wide, cleft 0.27 of its length, each lobe with two setae on surface and two distal spines.

##### Description of paratype male

(IZCAS-I-A1602-2), 6.3 mm.

**Head.** *Antenna II* (Fig. 7A): peduncle articles 3–5 in length ratio 1.0 : 2.6 : 3.0, with setae along anterior and posterior margins; flagellum with six articles, with spines and setae on first article and with setae on the rest articles; calceoli of crangonyctid type present on peduncular article 5 and first two flagellum articles; rod-like structures accompanied with setae on flagellum articles.

**Pereon.** *Gnathopod I* (Fig. 6A, B): coxal plate bearing three setae on anterodistal corner, 1.6 times as wide as deep; basis with long setae on posterior margin, anterior margin bare; merus bearing setae on posterodistal corner; carpus 0.8 times as long as wide, approximately 0.5 times as long as propodus, bearing five clusters of setae along posterior margin and two pectinate setae on posterodistal corner; propodus pear-shaped, palm margin with 14 distally notched spines; dactylus with one seta on anterior margin.

*Gnathopod II* (Fig. 6C, D): coxal plate bearing five setae on distal margin; basis with long setae on posterior margin, anterior margin bare; merus bearing setae on posterodistal corner; carpus 1.5 times as long as wide, approximately 0.7 times as long as propodus, bearing seven clusters of setae along posterior margin; propodus stout, palm margin with 12 distally notched spines; dactylus with one seta on anterior margin.

*Pereopods III–VII* (Fig. 7B–F): similar to those of male.

*Pereonites I*–*VI* without armature on dorsal margin. *Pereonite VII* (Fig. 7H): with seven setae on dorsal margin.

**Pleon.** *Pleonites I*–*III* (Fig. 7I–K): dorsal margins with five, two, and nine setae, respectively.

**Urosome.** *Urosomites* (Fig. 7L, M): urosomite I with four setae on dorsal margin; urosomite II with two spines on each side.

*Uropods I–III* (Fig. 5K–M): uropod I peduncle with one basofacial spine, with three spines on exterior side, interior and exterior distal corners with one spine respectively; inner ramus approximately 0.73 times as long as peduncle, with two spines on interior side, two simple setae on exterior side, and six terminal spines accompanied by one seta; outer ramus approximately 0.8 times of inner ramus, with two spines on exterior side and five terminal spines. Uropod II shorter, peduncle bearing one spine on exterior side and one spine on each distal corner; inner ramus with two spines on each side, distal part with two serrated and four simple robust spines accompanied by one seta; outer ramus approximately 0.72 times of inner ramus, with one spine on exterior side and five terminal spines. Uropod III peduncle 0.31 times as long as outer ramus, with one dorsal and three ventral robust spines; inner ramus absent; outer ramus bi-articulate, first article of outer ramus with three groups of stiff spines on interior and exterior sides, terminal article 0.18 times of the first article, with three distal setae, a little shorter than adjacent spines.

*Telson* (Fig. 7G): 1.2 times as long as wide, cleft 0.24 of its length, each lobe with two setae on surface and two distal spines accompanied by one short seta.

##### Habitat.

This species was collected from groundwater flowing through a cave of the Wulongdong National Forest Park.

##### Remarks.

*Pseudocrangonyx elegantulus* sp. n. is clustered with *Pseudocrangonyx yezonis* Akatsuka & Komai, 1922 supported by high statistical supports in the molecular phylogenetic tree. Unfortunately, the original description of the latter species is poor and no redescription has been published. The following comparisons are based on recent observations (Ko Tomikawa pers. comm.). The new species is morphologically similar to *Pseudocrangonyx yezonis* in antenna II with calceoli; the armature of gnathopods I and II and pereopods III–VII; both rami of pleopods with more than five articles; urosomite III dorsal margin without armature. It can be distinguished from *Pseudocrangonyx yezonis* Akatsuka & Komai, 1922 by the following characters (*Pseudocrangonyx yezonis* in parentheses): pereonites I–VI without armature on dorsal margin, only pereonite VII with dorsal setae (with long setae on dorsal margins of pereonites I–VII); uropod III terminal article of outer ramus a little shorter than adjacent spines (subequal).

The new species is most similar to *Pseudocrangonyx cavernarius* Hou & Li, 2003 in the armature of gnathopods I and II and pereopods III–VII; epimeral plate I without armature on distal margin; both rami of pleopods with more than five articles. The new species can be distinguished from *Pseudocrangonyx cavernarius* Hou & Li, 2003 by the following characters (*Pseudocrangonyx cavernarius* in parentheses): antenna II with calceoli (absent); inner plate of maxilla II with four plumose facial setae in an oblique row (with five plumose setae); urosomite I with two setae on dorsal margin (with four clusters of setae); urosomite III dorsal margin without armature (with one pair of fine spines); outer ramus of uropod I with five terminal spines (with four terminal spines); uropod II inner ramus with six terminal spines accompanied by one seta (with five terminal spines) and outer ramus with five terminal spines (with three terminal spines); uropod III peduncle with one dorsal and three ventral robust spines (with three distal spines), terminal article of outer ramus a little shorter than adjacent spines (longer); each lobe of both male and female telson with two setae on surface (with no armature).

The new species is similar to *Pseudocrangonyx asiaticus* Uéno, 1934, which was redescribed by Uéno (1966), in the accessory ﬂagellum being subequal to the first article of primary flagellum; the armature of gnathopods I and II and pereopods III–VII. It can be distinguished from *Pseudocrangonyx asiaticus* Uéno, 1934 by the following characters (*Pseudocrangonyx asiaticus* in parentheses): antenna II with calceoli (absent); incisor of mandible with five teeth (with 5–6 teeth); mandible spine row with five serrated spines (with 8–10 serrated setae); maxilliped inner plate with three stout apical spines, two serrated setae, and five plumose setae (with five serrated spines and seven plumose setae); sternal gills absent (present on gnathopod II and pereopods III–IV); each lobe of both male and female telson with two setae on surface (with no armature).

The new species is similar to *Pseudocrangonyx elenae* Sidorov, 2011 in body length (longer than 6.0 mm); the armature of gnathopod I and II and pereopods III–VII; epimeral plate I without armature on distal margin; both rami of pleopods with more than five articles; urosomite III dorsal margin without armature; terminal article of outer ramus of uropod III shorter than adjacent spines. It can be distinguished from *Pseudocrangonyx elenae* Sidorov, 2011 by the following characters (*Pseudocrangonyx elenae* in parentheses): accessory ﬂagellum of antenna I subequal to the first article of primary flagellum (shorter than accompanying flagellar article); antenna II of female with calceoli (absent); mandible spine row with five serrated spines (with six serrated setae); maxilla I with four plumose setae on inner plate (with five plumose setae); inner plate of maxilla II with four plumose facial setae in an oblique row (with five plumose setae); inner plate of maxilliped with three stout apical spines, two serrated setae, and five plumose setae (with five simple strong apical setae and nine plumose setae); epimeral plate II with two spines on distal margin (with one seta).

The new species resembles *Pseudocrangonyx gudariensis* Tomikawa & Sato, 2016 in epimeral plate I without armature on distal margin; urosomite III dorsal margin without armature. It can be distinguished from *Pseudocrangonyx gudariensis* Tomikawa & Sato, 2016 by the following characters (*Pseudocrangonyx gudariensis* in parentheses): accessory ﬂagellum of antenna I subequal to the first article of primary flagellum (longer); antenna II of female with calceoli (absent); mandible spine row with five serrated spines (with 2–3 weakly pectinate setae); maxilla I with four plumose setae on inner plate (with three plumose setae); inner plate of maxilla II with four plumose facial setae in an oblique row (with three plumose setae); inner plate of maxilliped with three stout apical spines, two serrated setae, and five plumose setae (with three apical and two subapical robust setae); terminal article of uropod III outer ramus a little shorter than adjacent spines (longer); epimeral plates II and III with two spines on distal margins (with one seta); telson of male cleft 0.24 of its length (0.08).

The new species is also similar to *Pseudocrangonyx holsingeri* Sidorov & Gontcharov, 2013 in the armature of gnathopod I and II and pereopods III–VII; epimeral plate I without armature on distal margin; both rami of pleopods with more than five articles. It differs from *Pseudocrangonyx holsingeri* Sidorov & Gontcharov, 2013 by the following characters (*Pseudocrangonyx holsingeri* in parentheses): accessory ﬂagellum of antenna I subequal to the first article of primary flagellum (longer); inner plate of maxilliped with three stout apical spines, two serrated setae, and five plumose setae (with two apical and three sub-apical setae); epimeral plate III with two spines on distal margin (with three setae); uropod I peduncle with one basofacial spine (with two basofacial spines in female); uropod III peduncle with one dorsal and three ventral robust spines (with two sets of stiff setae on distal margins).

Distinguishing features of all the 22 species of genus *Pseudocrangonyx* can be found in the key below.

### Molecular phylogeny

**Figure 8.**
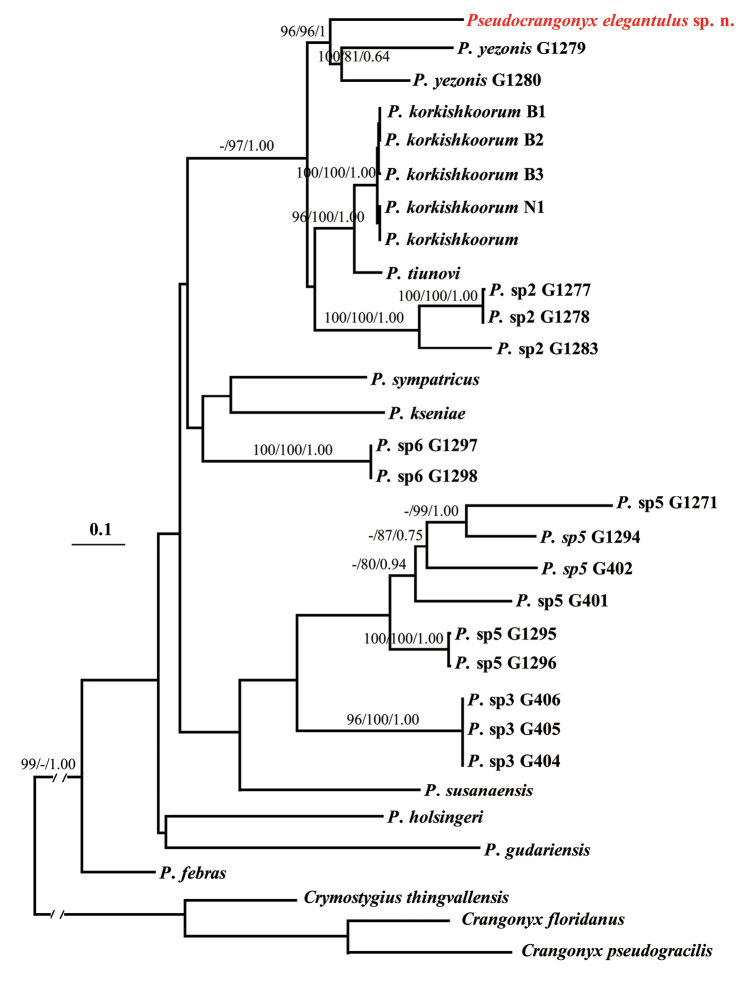
The ML tree derived from concatenated data set of 28S and COI. Support values greater than 70% are shown above branches in order for MP, ML, and BI analyses. Names of terminal taxa include voucher numbers for ingroups according to literature (Tomikawa et al. 2016).

The final alignment contained 32 taxa with 2123 bp, including 1465 bp for 28S and 658 bp for COI. MP, ML and BI yielded a congruent topology (Fig. 8). The monophyly of the genus *Pseudocrangonyx* was well supported, but the relationships within the genus *Pseudocrangoyx* remained unresolved, as found in the previous molecular study (Tomikawa et al. 2016). The new species *Pseudocrangonyx elegantulus* was clustered with *Pseudocrangonyx yezonis* with high support value. The uncorrected *p*-distance between *Pseudocrangonyx elegantulus* and *Pseudocrangonyx yezonis* was 12–15% for COI, which was comparable to distances found between Russian *Pseudocrangonyx* species (Sidorov and Gontcharov 2013).

As mentioned in the Remarks, the new species *Pseudocrangonyx elegantulus* is morphologically similar to *Pseudocrangonyx cavernarius*. Unfortunately, we were unable to obtain fresh material for *Pseudocrangonyx cavernarius*, because of tourist development in the type locality. Our phylogenetic results revealed that *Pseudocrangonyx elegantulus* was grouped with *Pseudocrangonyx yezonis* from the northern part of Japan. However, the divergences of 12–15% for COI confirmed the distinctness of new species, in comparison with the various COI distances used for amphipod delimitation (Rock et al. 2007). In addition, *Pseudocrangonyx elegantulus* and *Pseudocrangonyx yezonis* are mutually allopatric, as *Pseudocrangonyx elegantulus* is located at the inner land of China and separated from *Pseudocrangonyx yezonis* by sea. Therefore, morphological examination, molecular phylogenetic analyses, and distribution data support *Pseudocrangonyx elegantulus* being a new species.

### Key to the species of *Pseudocrangonyx*

**Table:** 

1	Epimeral plates II–III with sub-angled posterodistal corners	**2**
–	Epimeral plates II–III with obtuse or rounded posterodistal corners	**3**
2	Inner plate of maxilla I with five or more setae	**4**
–	Inner plate of maxilla I with less than five setae	**5**
3	Uropod I, ratio of outer ramus to inner ramus less than 0.5	***Pseudocrangonyx kyotonis* Akatsuka & Komai, 1922**
–	Uropod I, ratio of outer ramus to inner ramus higher than 0.5	**6**
4	Telson cleft 0.17 of its length	***Pseudocrangonyx bohaensis* (Derzhavin, 1927)**
–	Telson cleft less than 0.17 of its length	***Pseudocrangonyx yezonis* Akatsuka & Komai, 1922**
5	Mandible palp, article 3 equally long as article 2	***Pseudocrangonyx relicta* Labay, 1999**
–	Mandible palp, article 3 longer than article 2	***Pseudocrangonyx camtschaticus* Birstein, 1955**
6	Mandible palp, article 2 twice as wide as article 3	***Pseudocrangonyx birsteini* Labay, 1999**
–	Mandible palp, article 2 a little wider than article 3	**7**
7	Telson cleft more than or equal to 0.2 of its length	**8**
–	Telson cleft less than 0.2 of its length or not cleft	**9**
8	Maxilliped palp, article 3 less than 0.5 times as wide as deep	**10**
–	Maxilliped palp, article 3 higher than 0.5 times as wide as deep	**11**
9	Epimeral plates I–III with 7–9 setae on posterior margins	***Pseudocrangonyx manchuricus* Oguro, 1938**
–	Epimeral plates I–III with less than 9 setae on posterior margins	**12**
10	Maxilla I, inner plate with three plumose setae	***Pseudocrangonyx susanaensis* Labay, 1999**
–	Maxilla I, inner plate with more than three plumose setae	***Pseudocrangonyx asiaticus* Uéno, 1934**
11	Female antenna II with calceoli	***Pseudocrangonyx elegantulus* sp. n.**
–	Female antenna II without calceoli	**13**
12	Male gnathopod II armed with serrate robust setae at palmar angle	***Pseudocrangonyx febras* Sidorov, 2009**
–	Male gnathopod II armed with notched robust setae at palmar angle	**14**
13	Antenna I, accessory ﬂagellum subequal to first article of primary flagellum	***Pseudocrangonyx cavernarius* Hou & Li, 2003**
–	Antenna I, accessory ﬂagellum longer than first two articles of primary flagellum	***Pseudocrangonyx sympatricus* Sidorov & Gontcharov, 2013**
14	Antenna I, accessory ﬂagellum shorter than first article of primary flagellum	***Pseudocrangony levanidovi* Birstein, 1955**
–	Antenna I, accessory ﬂagellum longer than first article of primary flagellum	**15**
15	Female antenna II, flagellum with eight articles	**16**
–	Female antenna II, flagellum with less than eight articles	**17**
16	Uropod III, terminal article of outer ramus shorter than adjacent spines	***Pseudocrangonyx shikokunis* Akatsuka & Komai, 1922**
–	Uropod III, terminal article of outer ramus longer than adjacent spines	***Pseudocrangonyx korkishkoorum* Sidorov, 2006**
17	Maxilla I, inner plate with three plumose setae or less	**18**
–	Maxilla I, inner plate with more than three plumose setae	**19**
18	Telson not cleft	***Pseudocrangonyx kseniae* Sidorov, 2012**
–	Telson cleft	**20**
19	Female uropod I peduncle with two basofacial spines	***Pseudocrangonyx holsingeri* Sidorov & Gontcharov, 2013**
–	Female uropod I peduncle with one basofacial spine	**21**
20	Sternal gills absent	***Pseudocrangonyx gudariensis* Tomikawa & Sato, 2016**
–	Sternal gills present	***Pseudocrangonyx coreanus* Uéno, 1966**
21	Male antenna II with swollen peduncular article 5	***Pseudocrangonyx tiunovi* Sidorov & Gontcharov, 2013**
–	Male antenna II with a common peduncular article 5	***Pseudocrangonyx elenae* Sidorov, 2011**

## Discussion

Four *Pseudocrangonyx* species are recorded from subterranean freshwaters of China. *Pseudocrangonyx asiaticus* and *Pseudocrangonyx manchuricus* are known from interstitial water strata approximately 10 meters under the surface of the earth, while *Pseudocrangonyx cavernarius* and *Pseudocrangonyx elegantulus* inhabit caves. Because the subterranean habitats are imperiled by drought and tourism, conservation plans should be strengthened.

Our molecular analyses revealed significant COI differentiation (12–20%) for species of the genus *Pseudocrangonyx*. Molecular evidences help us to discover new species, especially for subterranean or cave species which are morphologically indistinguishable (Hou and Li 2010). Phylogenetic results supported a single origin of the genus *Pseudocrangonyx*, however the diversification pattern across the Asia-Pacific margins was uncertain. Extensive sampling and detailed genetic data are needed to clarify the evolutionary history of *Pseudocrangonyx* amphipods.

## Supplementary Material

XML Treatment for Pseudocrangonyx

XML Treatment for Pseudocrangonyx elegantulus
